# Effects of Cr Addition on the Microstructure and Mechanical Properties of an Al–Si–Cu–Mg Alloy

**DOI:** 10.3390/ma17143607

**Published:** 2024-07-22

**Authors:** Fengshan Sun, Xin Wen, Shuaifei Sun, Yuanyuan Lu, Wenlong Xiao, Chaoli Ma

**Affiliations:** 1 School of Materials Science and Engineering, Beihang University, Beijing 100191, China; by1201120@buaa.edu.cn (F.S.); sy2143225@buaa.edu.cn (X.W.); zy2101209@buaa.edu.cn (S.S.); wlxiao@buaa.edu.cn (W.X.); machaoli@buaa.edu.cn (C.M.); 2 Tianmushan Laboratory, Hangzhou 310023, China

**Keywords:** Al–Si–Cu–Mg alloy, Cr addition, heat treatment, mechanical properties, microstructure

## Abstract

The effects of chromium (Cr) addition ranging 0.1–0.3 wt.% on the microstructure and mechanical properties of Al–7Si–4Cu–0.25Mg (wt.%) alloy have been investigated. The cast Cr-free alloy consisted of α-Al, eutectic Si, Q-Al_5_Mg_8_Cu_2_Si_6_ and θ-Al_2_Cu phases. Doping of Cr resulted in the appearance of a polyhedron-shaped α-Al_13_Cr_4_Si_4_ phase with a cubic structure. The Al_13_Cr_4_Si_4_ particles were found to embed with Al_2_Cu blocks and bring about size reduction for the Al_2_Cu blocks. The area fraction of Al_13_Cr_4_Si_4_ monotonously increased with Cr content. After T6 treatment, the Al_2_Cu blocks almost fully dissolved and transformed to θ’-Al_2_Cu precipitates in the Cr-containing alloys. TEM observation revealed relatively large-sized θ’ precipitates attached to Al_13_Cr_4_Si_4_ dispersoids. The Cr-containing alloys showed impressive mechanical properties, with the peak strength up to 452 MPa at room temperature. The ductility exhibited an increasing trend with Cr content, but the strength dropped dramatically when the Cr content reached 0.3 wt.%. It is suggested that the strength contribution from the Al_13_Cr_4_Si_4_ phase is limited, especially at an elevated temperature.

## 1. Introduction

Al–Si-based alloys are attractive structural materials in the transport industry because of their high strength-to-weight ratio, good castability and low processing cost [1,2,3,4]. The mechanical properties of Al–Si based alloys have been remarkably improved over the past several decades by the means of microalloying. There have been several beneficial alloying elements identified, including Ni, Zr, Ti, Sr, Cr, etc. [5,6,7,8,9,10,11,12]. For example, morphological modification of eutectic Si from coarse plate-like segments to fine fibrous networks by alloying with Sr is a well-known path to improve mechanical performance for Al–Si alloys [9]. Alloying with Zr or Sc can generate Al_3_Zr or Al_3_Sc particles and bring about grain refinement for α-Al matrix, thereby increasing strength [10,11].

Among the above-mentioned alloying elements, Cr has been widely recognized as a modifier to optimize the morphology of Fe-rich intermetallic phases, i.e., from the acicular β-Al_5_FeSi phase to the script-like α-Al(Fe, Cr)Si phase. It is helpful for improving the ductility of Al–Si based alloys since stress concentration around the Fe-rich particles can be relieved [13,14]. Mahta et al. explained that the Cr atoms acted as additional Fe, converting the monolithic β-Al_5_FeSi phase to the α-Al(Fe, Cr)Si phase with hexagonal structure (close to cubic structure) and avoiding preferential growth along certain directions during solidification [14]. Recently, microalloying with Cr has received particular attention owing to its potentials in strengthening Al–Si based alloys [15,16]. Li et al. demonstrated that Cr addition could change the morphology of primary Al_15_(Mn, Cr)_3_Si_2_ phase and enhance its thermal stability, by which the high-temperature strength of an Al–Si–Cu–Mn alloy was improved [15]. The α-Al(Fe, Cr)Si phase resulting from a simultaneous addition of Cr and Fe was able to integrate with the existing Ni-rich phases and played a supportive strengthening role for a piston alloy (Al–Si–Cu–Mg–Ni–Fe) at 350 °C [16]. Nevertheless, reduction of ultimate tensile strength (UTS) relating to Cr addition at 350 °C has been reported by Yang et al. for another piston alloy [17]. They declaimed that the β-Al_5_FeSi phase should be stronger than the α-Al(Fe, Cr)Si phase at elevated temperatures because of the larger Si content. In this regard, the effects of Cr addition on the mechanical performance of Al–Si-based alloys are considerably complicated. 

It is noted that most of previous studies concerning Cr addition have focused on the combined effects of Cr with several other alloying elements, as evidenced by α-Al(Fe, Cr)Si, Al_15_(Mn, Cr)_3_Si_2_, Al(Fe, Mn, Cr)Si, Al_13_(Fe, V, Cr)_4_Si_4_ and other Cr-bearing intermetallic phases reported [6,12,15,16,17]. A recent work from Zhan and Hu revealed the appearance of a Cr-rich phase structurally similar to Al_13_Cr_4_Si_4_ in a 0.25 wt.% Cr-containing Al–Si–Cu–Mg alloy [18]. However, there have been no works examining the microstructure evolution or changes in mechanical properties as a function of Al_13_Cr_4_Si_4_ dispersoids. In other words, the individual effects of Cr are still not well understood.

This study is an attempt to investigate the effects of Cr addition on the microstructure and mechanical performance of an Al–Si–Cu–Mg alloy, aiming to provide insights on the optimal Cr content for alloy design. The content of Fe has been restricted to diminish possible effects from Fe-rich intermetallic phases. Both the as-cast and heat-treated alloys were carefully analyzed in terms of microstructure features and mechanical properties to comprehensively evaluate the effects of Cr addition. 

## 2. Materials and Methods

The base alloy (Al–7Si–4Cu–0.25Mg, wt.%) and Cr-containing alloys (0.1–0.3 wt.% Cr added) were prepared using high-purity Al (99.95%), Cu (99.9%), Mg (99.9%), Si (99.98%) and Al–10Sr, Al–5Cr, Al–5Ti–1B master alloys. All raw materials were melted in an electrical resistance furnace. The molten melt was degassed for ~30 min using pure argon, which was injected via a rotary degassing impeller. Each melt was held at 725 °C for 15 min and then poured into a permanent mold preheated to 150 °C. The actual chemical compositions of the alloy samples as measured by a thermal scientific ARL3460 OES melt analyzer are shown in Table 1. Following the works from Aguilera Luna and Sokolowski [19,20], a two-stage solution treatment consisting of a first stage at 500 °C for 45 min and a second stage at 520 °C for 4 h was performed. When the two-stage solution heat treatment was finished, the samples were quenched into hot water (~95 °C) and artificially aged at 170 °C for 5 h. 

Metallographic specimens were grounded using silicon carbide papers and polished to obtain a mirror-like surface. They were subsequently etched in a Keller reagent (1.5%HCL + 2.5%HNO_3_ + 1%HF + 95%H_2_O). A Thermo Fisher Apreo scanning electron microscope (SEM) (Waltham, MA, USA) equipped with energy dispersive spectroscopy (EDS) was employed for microstructure characterization and chemical analysis of constituent phases. An FEI Tecnai G2-F20 transmission electron microscope (TEM) (Waltham, MA, USA) was used for a more delicate observation. TEM specimens were firstly ground to a thickness of ~100 μm and then cut into small disks with the diameter of 3 mm. Afterward, fine polishing was conducted to reduce the thickness to ~50 μm. A Gatan 691 precision ion polishing system (Pleasanton, CA, USA) was finally applied to obtain high quality transparent areas.

Mechanical properties of the heat-treated alloys were evaluated by tensile tests at room temperature and 250 °C, respectively. The specimens for tensile tests were dog-bone shaped with the gauge length of 44 mm, width of 15 mm and thickness of 2 mm. Room temperature tensile tests were conducted using a SANS EUT5105 mechanical testing machine (Shanghai, China) at a strain of 1 × 10^−3^ s^−1^. An Instron 5966 mechanical testing machine (Norwood, MA, USA) was used for high temperature tensile tests and the corresponding strain rate was 2 × 10^−4^ s^−1^. The specimens were held at 250 °C for 5 min prior to testing. At least three specimens were tested for each alloy to calculate the average value of strength and elongation. 

## 3. Results

### 3.1. Microstructure Characteristics

**Figure 1:**
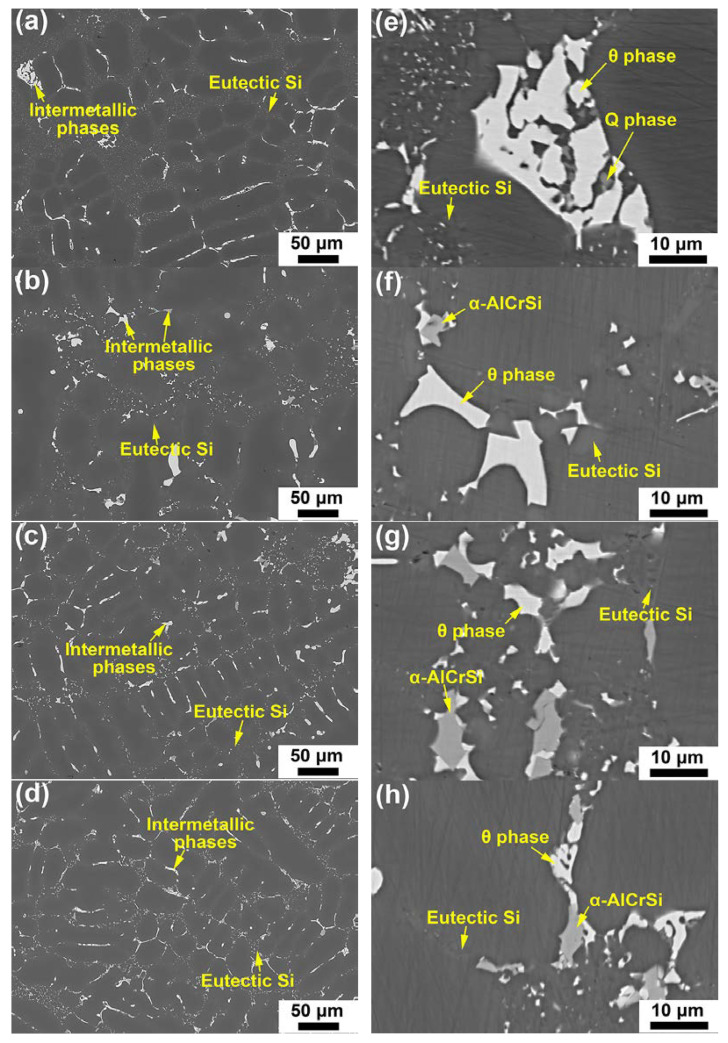
SEM-BSE micrographs of the (**a**,**e**) base, (**b**,**f**) Cr01, (**c**,**g**) Cr02 and (**d**,**h**) Cr03 alloys in as-cast state. The micrographs in (**a**–**d**) were captured at a low magnification while those in (**e**–**h**) were captured at a high magnification. These alloys are mainly composed of α-Al, eutectic Si, θ-Al_2_Cu, Q-Al_5_Mg_8_Cu_2_Si_6_ and a Cr-rich phase (indicated as α-AlCrSi).

Figure 1 displays back-scattered electron (BSE) micrographs of the base, Cr01, Cr02 and Cr03 alloys in the as-cast state. Figure 1a–d represents the micrographs captured at a low magnification while Figure 1e–h represents the micrographs captured at a high magnification. As seen in Figure 1, all the alloys consisted of α-Al dendrites (dark), eutectic Si particles (gray) and intermetallic compounds (bright). Both the Si particles and intermetallic compounds preferentially located at grain boundaries of the α-Al matrix. An enlarged view of the intermetallic compounds in the base alloy (Figure 1e) revealed that there were two types of intermetallic phases involved. EDS analysis (in Supplementary Materials) confirmed that the intermetallic phase with relatively bright contrast was θ-Al_2_Cu, and the relatively dark phase was Q-Al_5_Mg_8_Cu_2_Si_6_. Image analysis processed by ImageJ software (version 1.54a) indicates that the area fraction of Q phase in the base alloy was ~2.78%. However, the Q phase was barely observed in the Cr-containing alloys. Instead, a Cr-rich phase was detected, as pointed out in Figure 1f–h. This Cr-rich phase appeared as polyhedral blocks and embedded with the θ phase. Based on EDS results, the chemical compositions of the Cr-rich intermetallic compound in the Cr01, Cr02 and Cr03 alloys were similar to each other, i.e., Al~70.8%, Cr~11.3%, Si~11.1% and Cu~6.8% in atomic weight. It has been identified as the α-Al_13_Cr_4_Si_4_ phase containing a certain amount of Cu via TEM, which will be shown in following text. Table 2 summarizes the area fractions of major phase constituents for each sample. Apparently, the area fraction of the Cr-rich intermetallic phase increased with Cr content, indicating that increasing Cr concentration mainly gives rise to the increased amount of the Cr-rich intermetallic particles.

A noteworthy feature in Figure 1 is that the θ blocks of the Cr-containing alloys exhibited much finer than those of the base alloy. Quantitative analysis was performed by calibrating the length and width of the θ blocks, as shown in Table 3. The average length of the θ phase was 9.8 μm for the base alloy, while that for the Cr03 alloy was 6.3 μm. It was clearly revealed that the Cr addition promoted fragmentation of θ phase. This may be related to the appearance of the Cr-rich phase, which formed along with the θ phase and interrupted its continuous precipitation during solidification. In Table 3, the secondary dendrite arm spacing (SDAS) data are included. The SDAS values of the Cr02 and Cr03 alloys were 20.6 μm and 22.1 μm, respectively, which were slightly smaller than the SDAS of the base alloy (22.6 μm). Nevertheless, the SDAS of the Cr01 alloy was comparatively large (26.9 μm), suggesting that an Cr addition less than 0.1 wt.% might be insufficient in refining matrix grains.

**Figure 2:**
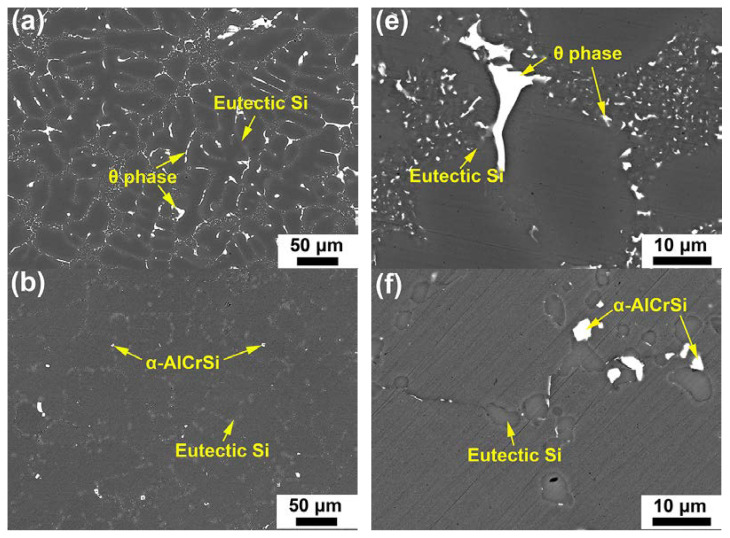

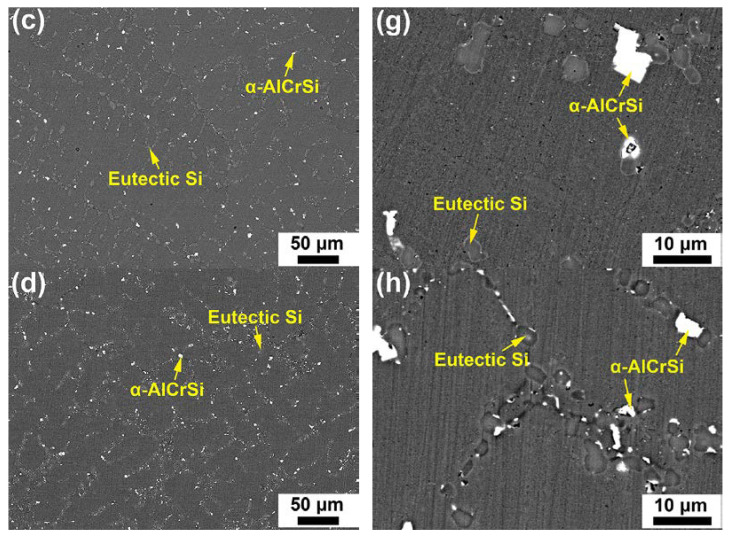
SEM-BSE micrographs of the (**a**,**e**) base, (**b**,**f**) Cr01, (**c**,**g**) Cr02 and (**d**,**h**) Cr03 alloys in heat-treated state. The micrographs in (**a**–**d**) were captured at a low magnification while those in (**e**–**h**) were captured at a high magnification.

Typical BSE micrographs as collected from the heat-treated alloys are presented in Figure 2. The T6 heat treatment applied induced obvious microstructural changes for the investigated alloys. As seen in Figure 2e–h, the eutectic Si particles became spheroidized, particularly in the Cr-containing alloys. Another change was manifested by the intermetallic phases remaining in the microstructure after heat treatment. In the case of base alloy, the Q phase disappeared but the θ phase was not fully dissolved (Figure 2e). However, the large-sized θ blocks could hardly be found in the Cr-containing alloys (Figure 2b–d). EDS examination verified the chemical composition of the intermetallic phase existing in the heat-treated Cr-containing alloys as Al~71.9%, Cr~11.2%, Si~10.9% and Cu~6.0% in atomic weight, which was nearly identical to that of the Cr-rich phase in their cast counterparts. The reduced size of the θ blocks associated with Cr addition could be a major reason responsible for the facilitated dissolving process of them during the solution heat treatment.

**Figure 3:**
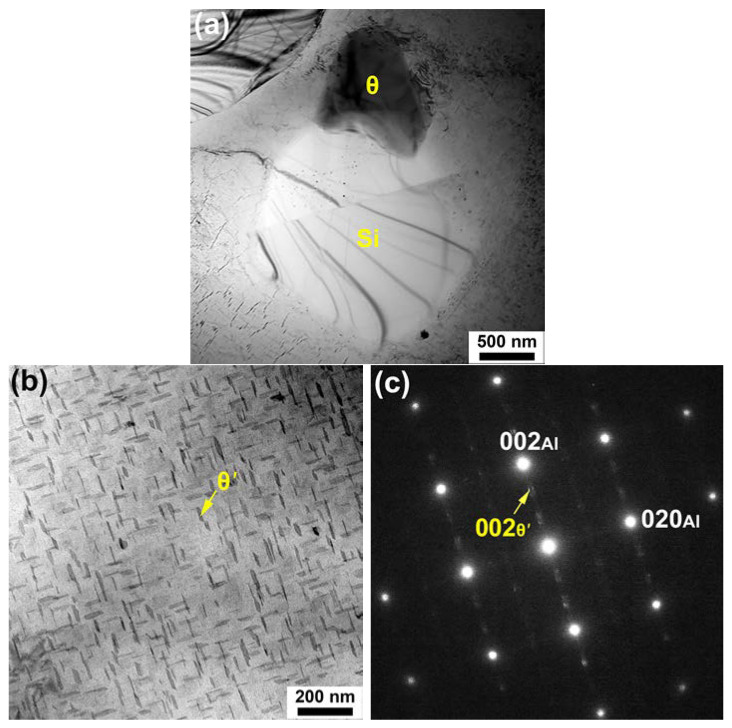
Bright-field (BF) TEM micrographs of the heat-treated base alloy captured at (**a**) a low magnification and (**b**) at a high magnification, with the diffraction pattern corresponding to (**b**) shown in (**c**).

**Figure 4:**
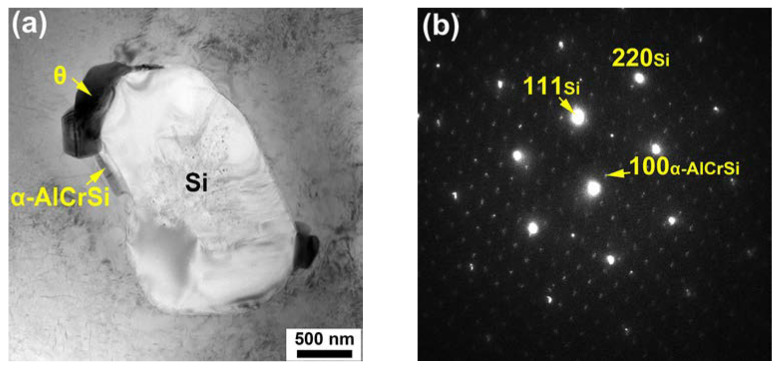
(**a**) BF-TEM micrograph of the heat-treated Cr02 alloy with the diffraction pattern obtained at the boundary between the Cr-rich phase and Si particle shown in (**b**).

**Figure 5:**
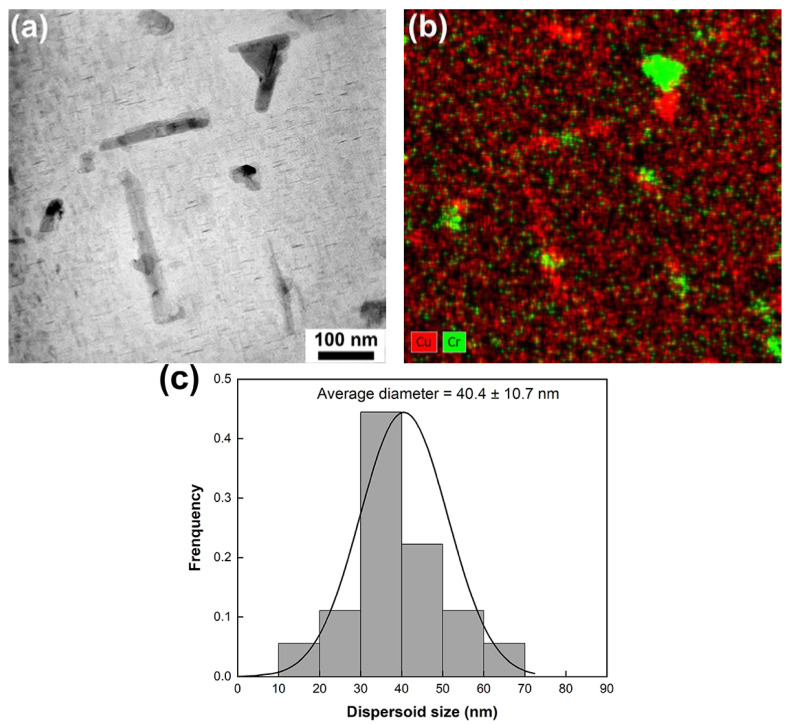
(**a**) BF-TEM micrograph of the heat-treated Cr02 alloy with corresponding elemental mappings of Cr (highlighted in green) and Cu (highlighted in red) shown in (**b**), (**c**) size distribution of Al_13_Cr_4_Si_4_ dispersoids as analyzed from 10 TEM micrographs of different regions.

Figure 3a,b are bright-field (BF) TEM micrographs obtained from the heat-treated base alloy at a relatively low magnification and a high magnification, respectively. The selected-area diffraction pattern (SADP) corresponding to Figure 3b is given in Figure 3c. Though small Al_2_Cu blocks were occasionally observed in the vicinity of Si particles, θ’-Al_2_Cu precipitates spread over the matrix. Figure 4a presents a BF-TEM micrograph of the heat-treated Cr02 alloy, in which Cr-rich dispersoids are clearly seen. The SADP captured at the boundary between the Cr-rich phase and Si particle is shown in Figure 4b, with selected diffraction spots indexed. Analyses on the diffraction patterns of at least five Cr-rich dispersoids indicate that all of them possessed a cubic structure with the lattice constant *a* of ~1.25 nm. Referring to the works from Robinson and Gustafsson [21,22], this phase was deduced to be α-Al_13_Cr_4_Si_4_. It is interesting to note that a small amount of Cu (~5.6 at.%) was also detected in the Al_13_Cr_4_Si_4_ dispersoids by TEM-EDS, in accordance with the SEM-EDS results. To better illustrate the distribution of Cr, EDS elemental mappings are provided in Figure 5. Figure 5a is a BF-TEM micrograph of the heat-treated Cr02 alloy obtained at a relatively high magnification and the corresponding elemental mappings of Cu and Cr are displayed in Figure 5b. Cu enrichment as highlighted in red color reflects θ’ precipitates, while Cr enrichment as highlighted in green color reflects Al_13_Cr_4_Si_4_ dispersoids. We can see that the Al_13_Cr_4_Si_4_ dispersoids were attached to the rod-like θ’ precipitates. These rod-like θ’ precipitates exhibited much larger than regular θ’ precipitates. It is highly possible that the Al_13_Cr_4_Si_4_ dispersoids acted as heterogenous nucleation sites for the θ’ precipitates during aging and prompted its fast growth to a fairly large size due to the high content of Cu in Al_13_Cr_4_Si_4_. Meanwhile, precipitate-free zones (PZFs) were observed around the θ’ rods. The PZFs surrounding Al_2_Cu rods have been seldom reported but Zhan and Hu have observed similar PZFs surrounding Mg_2_Si rods in an Al–Si–Mg–Cr alloy [18]. They demonstrated that the Al_13_Cr_4_Si_4_ dispersoids acted as nucleation sites for Mg_2_Si during quenching and the PZFs primarily resulted from the depletion of Mg and Si. Figure 5c is the size distribution histogram of the Al_13_Cr_4_Si_4_ dispersoids by analyzing ten TEM images from different regions. The average size of the dispersoids, as estimated by equivalent diameter, was 40.4 μm.

### 3.2. Mechanical Properties

**Figure 6:**
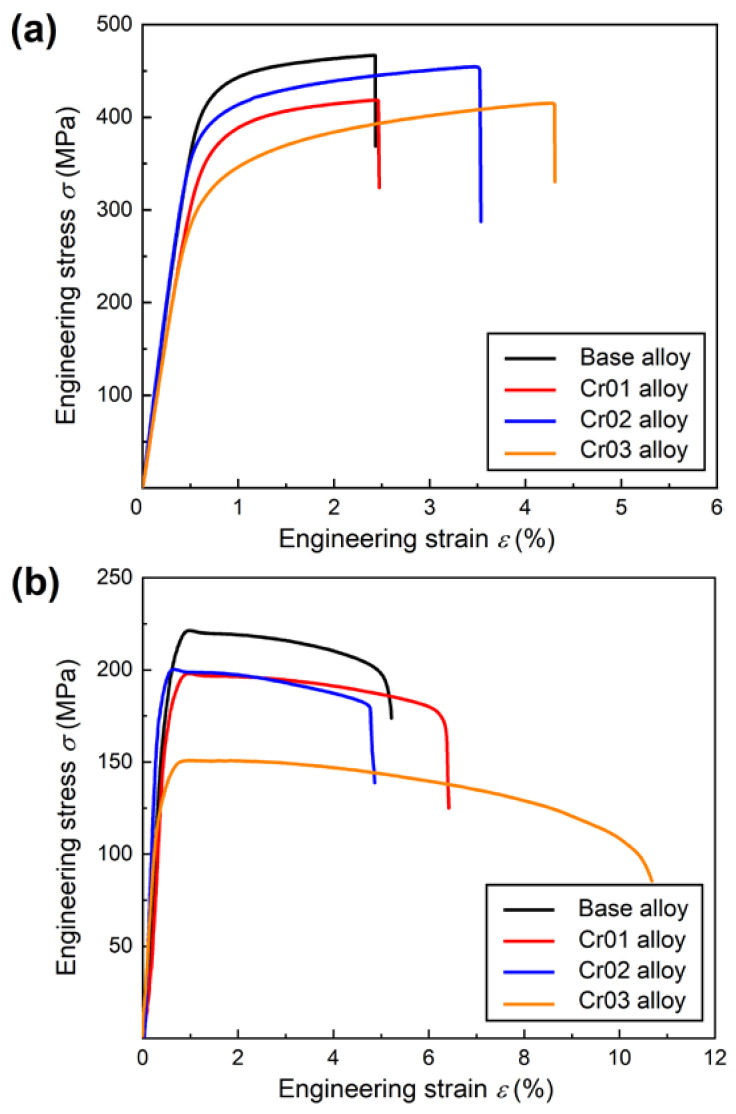
Engineering stress-strain curves of the heat-treated alloys tested at (**a**) room temperature and (**b**) 250 °C.

Figure 6a shows engineering stress-strain curves of the heat-treated alloys tested at room temperature. The extracted ultimate tensile strength (UTS), yield strength (YS, 0.2% offset stress) and elongation to fracture (EL) data are listed in Table 4. The base alloy exhibited impressive strength (UTS ~469 MPa), whereas its elongation was 2.3%. Adding 0.1 wt.% Cr did not improve the elongation and even brought about loss in strength (see Table 4). Balanced mechanical performance was achieved in the Cr02 alloy, with a UTS of 452 MPa and EL of 3.5%. The Cr03 alloy showed the lowest UTS (415 MPa) and the highest EL (4.8%) among all the samples. It was revealed that the ductility was roughly increasing with Cr content. Nevertheless, dramatic strength drop occurred when the Cr addition reached 0.3 wt.%. These variations of mechanical properties could be partly rationalized by the weakened strengthening effect from the θ’ precipitates, as accompanied with the appearance of Al_13_Cr_4_Si_4_.

The engineering stress-strain curves of the heat-treated alloys tested at 250 °C are plotted in Figure 6b. Alike the room temperature stress-strain responses, the highest strength level (UTS ~222 MPa) at 250 °C was obtained in the base alloy (Table 5). The lowest strength level (UTS ~155 MPa) and the highest EL (~10.2%) were observed in the Cr03 alloy. The inferior mechanical performance of the Cr-containing alloys implies that the strength contribution from the Al_13_Cr_4_Si_4_ dispersoids should be limited at elevated temperatures. 

### 3.3. Fractography

**Figure 7:**
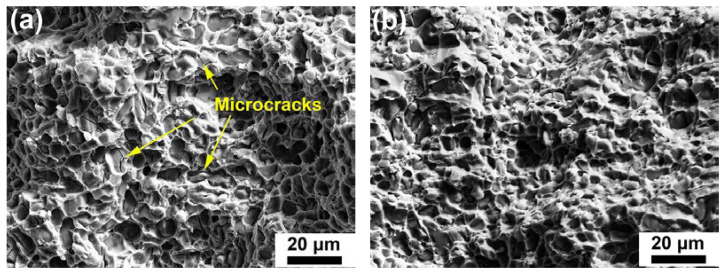
SEM micrographs illustrating the fracture surfaces of the heated-treated alloys after room temperature tensile tests, (**a**) base alloy and (**b**) Cr02 alloy.

**Figure 8:**
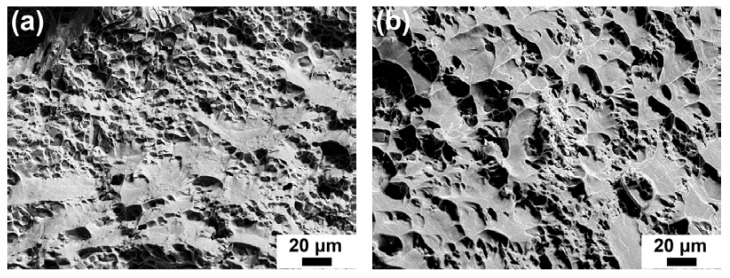

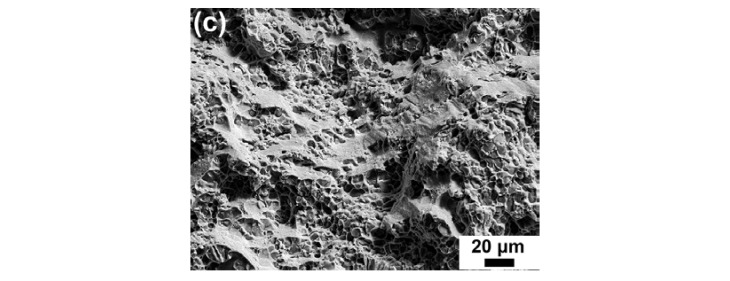
SEM micrographs illustrating the fracture surfaces of the heated-treated alloys after 250 °C tensile tests, (**a**) base alloy, (**b**) Cr02 alloy and (**c**) Cr03 alloy.

Fracture surfaces of the heat-treated base alloy and Cr02 alloy after room temperature tensile tests are shown in Figure 7. Both brittle and ductile fracture features are discernable, e.g., micro-cracks of the Si particles and intermetallic phases and tear ridges of the α-Al matrix. It is noticeable that the density of microcracks in the Cr02 sample (Figure 7b) was lower than that in the base alloy (Figure 7a). Correlating with the microstructure characteristics displayed in Figure 2, the reduced microcrack density of the Cr02 sample could be ascribed to the absence of large-sized θ blocks. Figure 8 represents the fracture surfaces of the heat-treated base alloy, Cr02 and Cr03 alloys after the 250 °C tensile tests. The fracture surfaces were overwhelmed by shear dimples [23], which agrees with the improved ductility of the alloy samples at 250 °C.

## 4. Discussion

### 4.1. Effect of Cr Addition on Microstructure

According to the Al–Cr binary phase diagram [24], the solubility of Cr in α-Al matrix is extremely low (i.e., ~0.04% at 540 °C), and thus, Cr-bearing intermetallic phases are expected to form in Al alloys. For most Al–Si based alloys, Cr is intentionally added to replace needle-like β-Al_5_FeSi phase with Chinese-script-like α-Al(Fe, Cr)Si phase, by which stress concentration upon loading can be relieved. Other Cr-bearing intermetallic phases, such as α-Al(Fe, Mn, Cr)Si and Al_13_(Fe, V, Cr)_4_Si_4_, have also been reported in compositionally complex alloys [6,15,16]. It was demonstrated that Cr preferentially dissolves into Al–Fe–Si phases through occupying the position of Fe atoms. These (Cr, Fe)-bearing intermetallic phases are generally recognized with cubic structures, e.g., the Al_15_(Fe, Cr)_3_Si_2_ phase with lattice constant *a* = 1.25 nm [25] and the Al_13_(Fe, Cr)_4_Si_4_ phase with *a* = 1.09 nm [26].

In this study, the content of Fe has been restricted to a negligible level (see Table 1). Therefore, the abovementioned (Cr, Fe)-bearing phases have not been detected in the Cr-containing alloys investigated. The experimental results revealed that doping Cr into the base alloy (Al–Si–Cu–Mg system) mainly led to the formation of Al_13_Cr_4_Si_4_ phase, and its amount progressively increased with Cr content. The crystallographic structure of Al_13_Cr_4_Si_4_ (space group F$\bar{4}$3m, lattice constant of 1.09 nm) was firstly reported by Robinson [21]. The Al_13_Cr_4_Si_4_ phase of the present work showed a larger lattice constant (*a* = 1.25 nm), which was possibly caused by the involvement of Cu. 

Comparing the microstructures of the Cr-free (base alloy) and Cr-containing alloys, the most obvious change associated with Cr addition was the morphology of Al_2_Cu phase. In as-cast state, the θ-Al_2_Cu blocks of the Cr-containing alloys appeared much finer than those of the base alloy. In heat-treated state, the Al_13_Cr_4_Si_4_ dispersoids were found to connect with the rod-like θ’-Al_2_Cu precipitates, which showed a larger size than regular θ’ precipitates. The Cu enrichment of the Al_13_Cr_4_Si_4_ dispersoids is proposed to account for the fast growth of θ’ precipitates around them. Local Cu enrichment around Al_13_Cr_4_Si_4_ dispersoids was also reported by An et al. in an Al–7.55Si–0.5Cu–0.58Mg–0.25Fe alloy [27]. It seems that Cr addition would disturb the uniform distribution of Cu and induce Cu enrichment in Al–Si–Cu–Mg alloys. 

### 4.2. Effect of Cr Addition on Mechanical Properties

As compared with other documented A319 type alloys [28,29,30], the investigated alloys, especially the Cr02 alloy, exhibited excellent strength and ductility at room temperature (see Table 4). It can be attributed to the exploit of the strengthening effect from intermetallic phases via appropriate heat treatment, as well as the elimination of harmful Fe-rich phases. 

To clarify the influence of Cr addition on the strength of Al–Si–Cu–Mg alloys, the strength data of the Cr-containing alloys will be theoretically elaborated. The base alloy was not considered here because of its distinct microstructure feature in heat-treated state (undissolved bulk Al_2_Cu phase). Regarding the limited solubility of Cr in the α-Al matrix, the strength contribution from solid solution hardening ($∆\sigma_{SS}$) should be insignificant. The strength contribution relating to SDAS reduction ($∆\sigma_{GB}$) can be estimated via the Hall–Petch law. Learned from Table 3, the SDAS decreased from 26.9 μm of the Cr01 sample to 20.6 μm of the Cr02 sample. Hence, the corresponding $∆\sigma_{GB}$ can be calculated as

(1) $$∆\sigma_{GB}=k\left({d_{Cr02}}^{-0.5}-{d_{Cr01}}^{-0.5}\right)$$

where *k* represents the H-P coefficient and is adopted as 0.326 MPa⸱m^−0.5^ by following the works of Ceschini and Wei [31,32]. The $\sigma_{GB}$ is then calculated to be ~8.9 MPa. By subtracting $∆\sigma_{GB}$ from the total increment of YS from the Cr01 sample to the Cr02 sample (~21 MPa), the strength contribution $\sigma_{P}$ arising from the θ’ precipitates and Al_13_Cr_4_Si_4_ dispersoids is derived, ~12.1 MPa. It is noted that the derived $\sigma_{P}$ is close to the strength increment resulting from 0.25 wt.% Cr addition to the Al–Si–Cu–Mg alloy (14 MPa) as evaluated by An et al. using the Orowan model [27]. 

Unfortunately, the strengthening effect of Al_13_Cr_4_Si_4_ dispersoids was not retained at a high Cr level (i.e., 0.3 wt.%) since a drastic strength drop was observed in the Cr03 alloy. It is inferred that the strength of the Al–Si–Cu–Mg alloy investigated is more dependent on the θ’ precipitates. The increased number density of the Al_13_Cr_4_Si_4_ dispersoids brought about a great deal of large θ’ rods at the cost of fine θ’ precipitates (Figure 5), which eventually impaired overall precipitation strengthening effect. Analogous strength reduction caused by the appearance of Al_13_Cr_4_Si_4_ dispersoids had been reported by An and coworkers [27], who attributed it to the depletion of Cu and a reduction in the volume of refined precipitates that could be achieved in aging treatment. 

**Figure 9:**
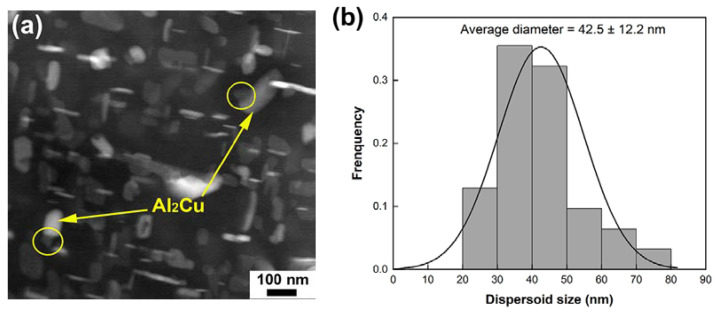
(**a**) Typical dark-field TEM image of the heat-treated Cr02 sample deformed at 250 °C, in which the Al_13_Cr_4_Si_4_ dispersoids (as circled out) were attached to coarsened θ’ precipitates (as pointed out by arrows), and (**b**) size distribution histogram of the Al_13_Cr_4_Si_4_ dispersoids.

High temperature strength plays a critical role in determining service temperature of Al–Si–Cu–Mg alloys. Above 250 °C, most traditional strengthening phases in these alloys, such as Al_2_Cu and Mg_2_Si, become coarsened and thus less powerful in strengthening. Introducing thermally stable phases, either in situ or ex situ, has been extensively applied to improve strength in this temperature regime. Al_13_Cr_4_Si_4_ has been proved to own outstanding thermal stability at 300 °C and is believed to be a promising candidate for strengthening Al–Si alloys at elevated temperatures [27]. However, the tensile test results of the present work demonstrate that the strength contribution from Al_13_Cr_4_Si_4_ dispersoids was quite weak at 250 °C. The inferior UTS of the Cr03 alloy even indicates that a large amount of Al_13_Cr_4_Si_4_ dispersoids could be detrimental for the strength. To further explore the effects of Al_13_Cr_4_Si_4_ on the high temperature strength of Al–Si–Cu–Mg alloys, the deformed microstructures of the Cr-containing alloys were examined by TEM. Figure 9 is a representative dark-field TEM micrograph of the deformed Cr02 sample after a 250 °C tensile test. It was found that the Al_13_Cr_4_Si_4_ dispersoids (as circled in Figure 9a) were still closely attached to the θ’ precipitates. The average size of the Al_13_Cr_4_Si_4_ dispersoids was measured to be ~42.5 μm, approaching the average Al_13_Cr_4_Si_4_ dispersoid size value of the undeformed Cr02 sample (Figure 5c). In contrast, the θ’ precipitates became significantly coarsened, which should be the main factor leading to the degraded strength. The moderate strength level of the Cr-containing alloys also indicates that the Al_13_Cr_4_Si_4_ phase might not be very strong at 250 °C. Despite good thermal stability, the undesirable high-temperature strength of Al_13_Cr_4_Si_4_, together with its insufficient capability in resisting the coarsening of θ’ precipitates, suggest it is not a favorable hardening phase for Al–Si–Cu–Mg alloys above 250 °C. 

Here, the reported strengthening effects of other Cr-bearing intermetallic phases for Al–Si based alloys at high temperatures are summarized. According to the work from Yang et al. [17], the strengthening effect of the α-Al(Fe, Cr)Si phase containing 70.18 at.% Al, 13.24 at.% Si, 9.3 at.% Fe and 7.28 at.% Cr was weaker than β-Al_5_FeSi at 350 °C. In contrast, the Al_15_(Mn, Cr)_3_Si_2_ phase containing 69.89 at.% Al, 13.73 at.% Si, 11.68 at.% Mn and 4.11 at.% Cr has been proved to offer a supportive strengthening role at 150 °C, 250 °C and 350 °C [15]. Moreover, Shaha et al. demonstrated the positive strengthening effect of the Al_13_(Fe, Cr, V, Ti)_4_Si_4_ phase (11.34 at.% Al, 43.42 at.% Si, 3.73 at.% Fe, 31.92 at.% Cr and 5.44 at.% V, 4.15 at.% Ti) and the Al_15_(Fe, Cr, V)_3_Si_2_ phase (63.72 at.% Al, 11.99 at.% Si, 10.87 at.% Fe, 11.06 at.% Cr, 2.36 at.% V) at 200 °C and 300 °C [33]. It is inferred that the (Mn, Cr)-bearing phases and (Mn, V, Cr)-bearing phases act stronger than the Cr-bearing phases and (Fe, Cr)-bearing phases in strengthening at elevated temperatures (e.g., ≥250 °C), which might be related to the diffusion rates of the involved elements. Therefore, a simultaneous addition with Mn or V is recommended to exploit the strengthening potentials of Cr.

At both room temperature and high temperature, the EL of the Al–Si–Cu–Mg alloy samples showed a straightforward increasing tendency with Cr content. The improved ductility, in accordance with the decreased strength, can also be rationalized by the diminished number density of the fine θ’ precipitates. Given this fact, manipulating Cr addition provides a feasible way to tailor mechanical performance for Al–Si–Cu–Mg alloys. Studies on the ductility and fracture mode of Al-based alloys (Al–Si–Cu, Al–Si–Cu–Mg and Al–Cu systems) with varying Cr and Cu contents will be carried out in our future work to achieve a thorough understanding of the competition between strengthening from Cu and toughening from Cr.

## 5. Conclusions

In this work, the variations of microstructure and mechanical properties with Cr addition ranging 0.1–0.3 wt.% have been systematically investigated for Al–7Si–4Cu–0.25Mg alloy. The main conclusions are as follows:

The Cr addition at all levels induced the formation of α-Al_13_Cr_4_Si_4_ phase, which was identified to be cubic-structured with the lattice constant of ~1.25 nm. The area fraction of Al_13_Cr_4_Si_4_ monotonously increased with Cr content.

Remarkable morphological changes of Al_2_Cu phase associated with the appearance of Al_13_Cr_4_Si_4_ were observed. In the as-cast state, polyhedron-shaped Al_13_Cr_4_Si_4_ particles embedded with θ-Al_2_Cu blocks and separated them into small pieces. After T6 treatment, the θ blocks were dissolved while θ’-Al_2_Cu precipitates widely distributed in the α-Al matrix. Large-sized θ’ precipitates in rod shape were found to connect with the Al_13_Cr_4_Si_4_ dispersoids.

The Cr-containing alloys showed impressive mechanical properties at a Cr concentration of 0.2 wt.%. The peak strength approached 452 MPa at room temperature and 199 MPa at 250 °C. Strength drop occurred when the Cr content reached 0.3 wt.%. The ductility exhibited an increasing trend with Cr addition.

## Figures and Tables

**Table 1:** Actual chemical compositions of the alloy samples.

Alloy	Element (wt.%)							
	Si	Cu	Fe	Mg	Cr	Ti	Sr	Al
Base	7.03	3.94	0.03	0.27	--	0.01	0.02	Bal.
Cr01	7.05	3.95	0.04	0.25	0.09	0.01	0.01	Bal.
Cr02	6.94	3.95	0.05	0.24	0.21	0.01	0.02	Bal.
Cr03	6.95	3.91	0.05	0.23	0.29	0.01	0.01	Bal.

**Table 2:** Areas fractions of phase constituents in the as-cast alloys.

Alloy	Eutectic Si	θ-Al_2_Cu	Q-Al_5_Mg_8_Cu_2_Si_6_	Cr-Rich Phase
Base	17.30 ± 0.32	4.47 ± 2.11	2.78 ± 0.81	--
Cr01	17.24 ± 2.09	1.92 ± 0.12	--	1.00 ± 0.10
Cr02	16.85 ± 1.27	2.85 ± 0.48	--	1.51 ± 0.14
Cr03	18.31 ± 1.18	1.33 ± 0.28	--	1.80 ± 0.38

**Table 3:** Geometric features of the θ-Al_2_Cu phase and SDAS for the as-cast base, Cr01, Cr02 and Cr03 alloys.

Alloy	Average Length of θ Blocks, μm	Average Width of θ Blocks, μm	SDAS, μm
Base	9.8 ± 3.7	3.6 ± 1.8	22.6 ± 1.6
Cr01	8.3 ± 3.7	3.7 ± 2.4	26.9 ± 2.8
Cr02	8.5 ± 4.0	3.8 ± 2.7	20.6 ± 2.1
Cr03	6.3 ± 3.4	2.8 ± 0.9	22.1 ± 3.1

**Table 4:** Mechanical properties of the heat-treated alloys at room temperature.

Alloy	UTS, MPa	YS, MPa	EL, %
Base	469 ± 1	426 ± 6	2.3 ± 0.4
Cr01	418 ± 4	370 ± 3	2.1 ± 0.4
Cr02	452 ± 5	391 ± 9	3.5 ± 0.1
Cr03	415 ± 3	322 ± 2	4.8 ± 0.7

**Table 5:** Mechanical properties of the heat-treated alloys at 250 °C.

Alloy	UTS, MPa	YS, MPa	EL, %
Base	222 ± 5	206 ± 6	5.8 ± 0.2
Cr01	197 ± 1	181 ± 1	6.1 ± 0.5
Cr02	199 ± 8	183 ± 4	4.8 ± 0.4
Cr03	155 ± 1	140 ± 1	10.2 ± 1.4

## Data Availability

Data are contained within the article and Supplementary Materials.

## Supplementary Materials

The following supporting information can be downloaded at: https://www.mdpi.com/article/10.3390/ma17143607/s1. Supplementary: EDS analysis of the as-cast and heat-treated alloy samples.
