# Supplementary material for: Effects of Cr Addition on the Microstructure and Mechanical Properties of an Al–Si–Cu–Mg Alloy
Source: Materials (Basel). 2024 Jul 22;17(14):3607. doi: 10.3390/ma17143607 (PMC11278795; doi:10.3390/ma17143607)
Supplement: Supplementary file 1 [file materials-17-03607-s001.zip › materials-3097764-supplementary.pdf]

Supplementary

1. SEM-EDS analysis of **the base alloy in as-cast state**

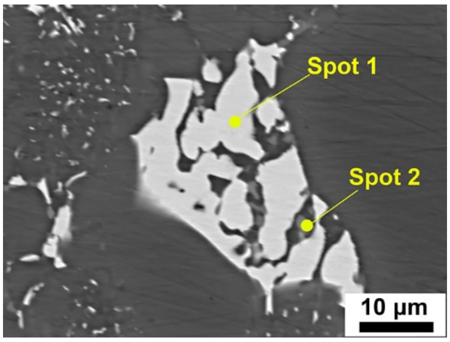

Spot 1

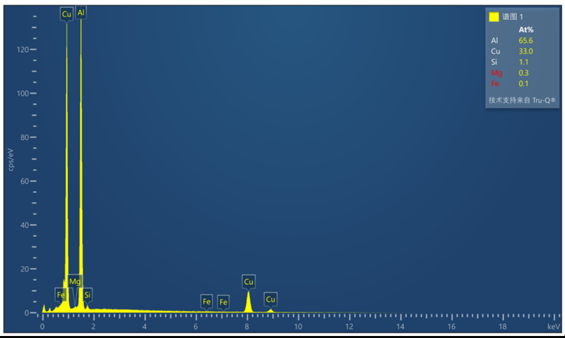

Element	Weight %	Atomic %
Mg	0.17	0.28
Al	45.26	65.55
Si	0.76	1.06
Fe	0.14	0.10
Cu	53.66	33.01

Spot 2

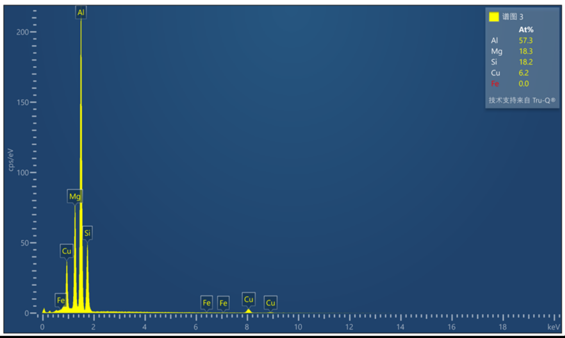

Element	Weight %	Atomic %
Mg	15.38	18.31
Al	53.39	57.30
Si	17.65	18.20
Fe	0.00	0.00
Cu	13.58	6.19

2. SEM- EDS analysis of **the Cr02 alloy in as-cast state**

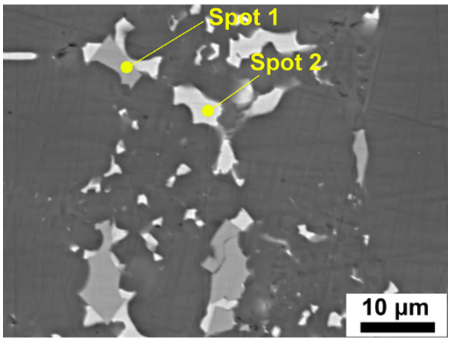

Spot 1

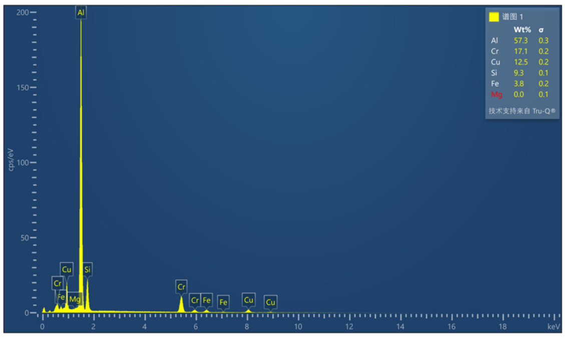

Element	Weight %	Atomic %
Mg	0.00	0.00
Al	57.33	69.70
Si	9.27	10.83
Cr	17.09	10.78
Fe	3.83	2.25
Cu	12.48	6.44

Spot 2

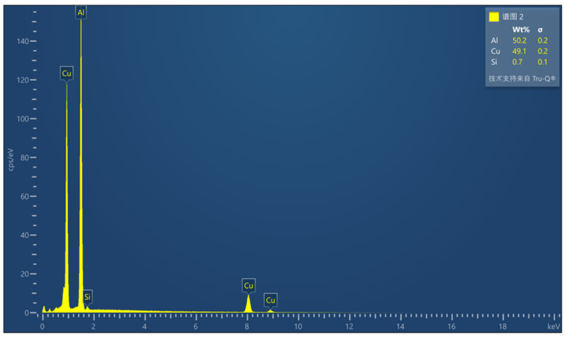

Element	Weight %	Atomic %
Al	50.19	69.97
Si	0.72	0.97
Cu	49.08	29.06

3. SEM-EDS analysis of **the base alloy in heat-treated state**

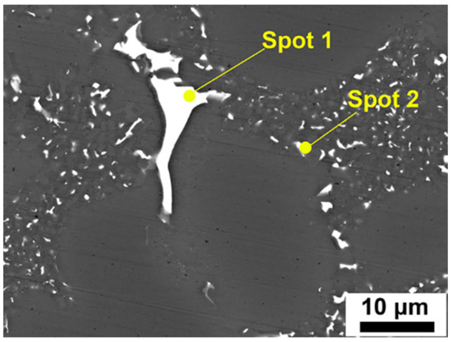

Spot 1

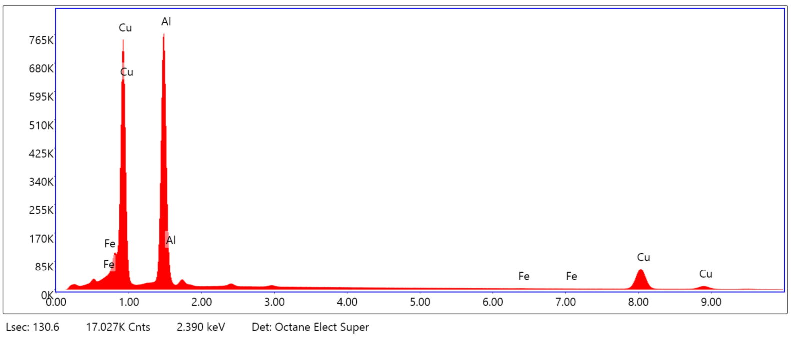

Element	Weight %	Atomic %
Al	49.20	69.51
Fe	0.05	0.03
Cu	50.75	30.46

Spot 2

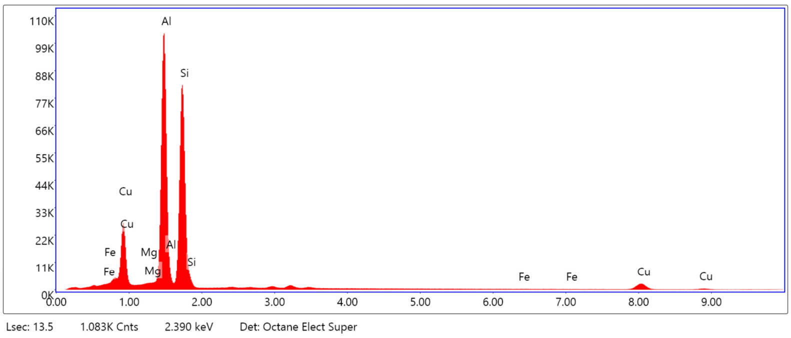

Element	Weight %	Atomic %
Mg	1.24	1.85
Al	47.89	64.20
Si	6.92	8.91
Fe	0.25	0.16
Cu	43.70	24.88

4. SEM-EDS analysis of **the Cr02 alloy in heat-treated state**

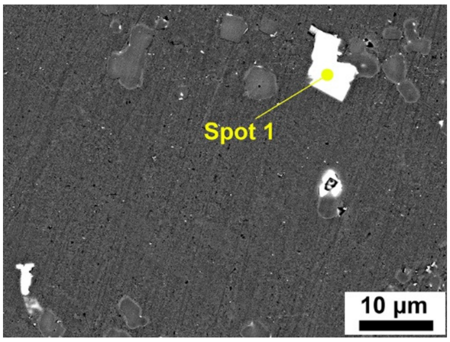

Spot 1

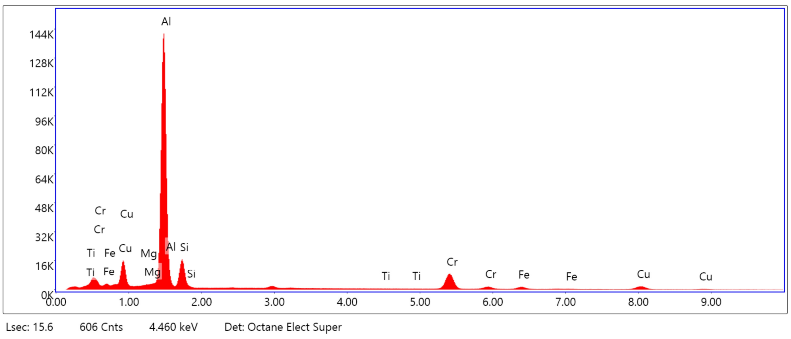

Element	Weight %	Atomic %
Mg	1.03	1.38
Al	57.49	69.15
Si	9.36	10.81
Ti	0.06	0.04
Cr	17.57	10.96
Fe	3.57	2.08
Cu	10.92	5.58

5. TEM-EDS analysis of **the base alloy in heat-treated state**

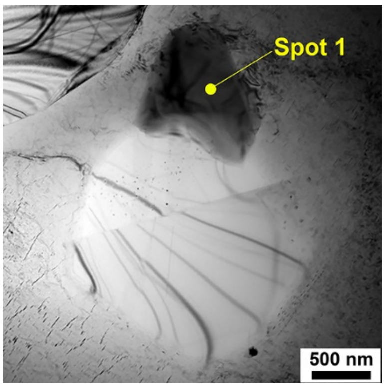

Spot 1

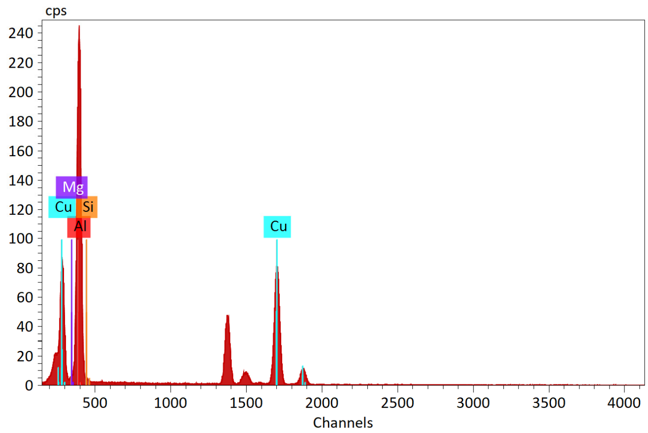

Element	Weight %	Atomic %
Mg	0.00	0.00
Al	48.34	68.57
Si	0.41	0.56
Cu	51.25	30.87

6. TEM-EDS analysis of **the C02 alloy in heat-treated state**

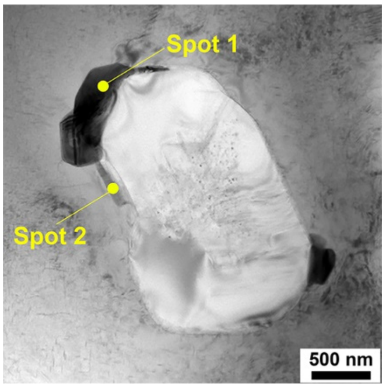

Spot 1

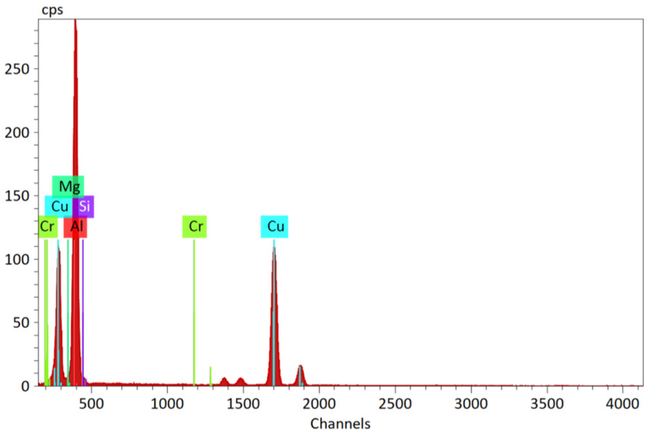

Element	Weight %	Atomic %
Mg	0.00	0.00
Al	45.18	65.62
Si	0.71	0.99
Cr	0.06	0.04
Cu	54.06	33.34

Spot 2

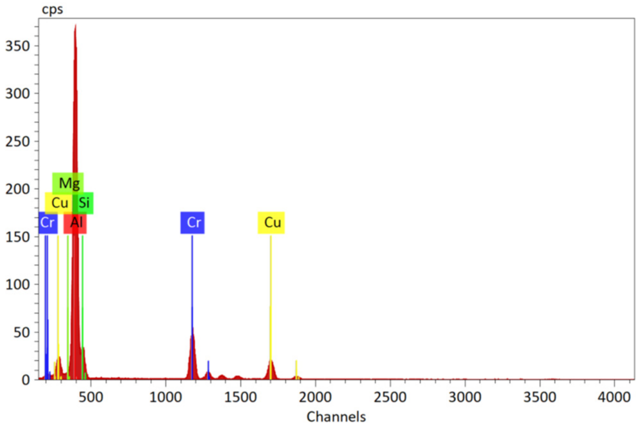

Element	Weight %	Atomic %
Mg	0.00	0.00
Al	66.01	77.47
Si	5.94	6.69
Cr	16.80	10.23
Cu	11.25	5.60
